# The Experience of 3D Total-Body Photography to Monitor Nevi: Results From an Australian General Population-Based Cohort Study

**DOI:** 10.2196/37034

**Published:** 2022-06-20

**Authors:** Caitlin Horsham, Montana O'Hara, Saira Sanjida, Samantha Ma, Dilki Jayasinghe, Adele C Green, Helmut Schaider, Joanne F Aitken, Richard A Sturm, Tarl Prow, H Peter Soyer, Monika Janda

**Affiliations:** 1 Centre for Health Services Research Faculty of Medicine The University of Queensland Brisbane Australia; 2 Dermatology Research Centre The University of Queensland Diamantina Institute The University of Queensland Brisbane, Queensland Australia; 3 Cancer and Population Studies QIMR Berghofer Medical Research Institute Brisbane, Queensland Australia; 4 Cancer Research UK Manchester Institute, University of Manchester Manchester Academic Health Sciences Centre Manchester United Kingdom; 5 Australian Skin and Skin Cancer Research Centre Brisbane, Queensland Australia; 6 Viertel Cancer Research Centre Cancer Council Queensland Brisbane, Queensland Australia; 7 Institute for Resilient Regions University of Southern Queensland Brisbane, Queensland Australia; 8 School of Public Health University of Queensland Brisbane, Queensland Australia; 9 Future Industries Institute University of South Australia Adelaide, South Australia Australia; 10 Skin Research Centre York Biomedical Research Institute Hull York Medical School York United Kingdom

**Keywords:** melanocytic nevi, melanoma, moles, skin cancer, early detection, 3D total-body photography, artificial intelligence, cohort study, skin, skin surface

## Abstract

**Background:**

Digital 3D total-body photography of the skin surface is an emerging imaging modality that can facilitate the identification of new and changing nevi.

**Objective:**

We aimed to describe the experiences of study participants drawn from the general population who were provided 3D total-body photography and dermoscopy for the monitoring of nevi.

**Methods:**

A population-based prospective study of adults aged 20-70 years from South East Queensland, Australia was conducted. Participants underwent 3D total-body photography and dermoscopy every 6 months over a 3-year period. Participants were asked to provide closed and open-ended feedback on their 3D total-body photography and dermoscopy experience (eg, comfort, trust, intended future use, and willingness to pay) at the halfway study time point (18 months) and final study time point (36 months). We assessed changes in participants’ reported experience of 3D total-body photography, and patient characteristics associated with patient experience at the end of the study (36 months) were analyzed.

**Results:**

A total of 149 participants completed the surveys at both the 18- and 36-month time points (median age 55, range 23-70 years; n=94, 63.1% were male). At the 18-month time point, most participants (n=103, 69.1%) stated they completely trusted 3D total-body imaging for the diagnosis and monitoring of their nevi, and this did not change at the 36-month (n=104, 69.8%) time point. The majority of participants reported that they were very comfortable or comfortable with the technology at both the 18- (n=138, 92.6%) and 36-month (n=140, 94%) time points, respectively; albeit, the number of participants reporting that they were very comfortable reduced significantly between the 18- and 36-month time points, from 71.1% (n=106) to 61.1% (n=91; *P*=.01). Almost all participants (n=140, 94%) would consider using this technology if it were to become commercially available, and this did not change during the two study time points. Half of the participants (n=74) cited barriers to participating in 3D total-body photography, including trust in the ability of this technology to detect and monitor suspicious lesions, digital privacy, cost, and travel requirements.

**Conclusions:**

The majority of participants expressed positive attitudes toward 3D total-body photography for the monitoring of their moles. Half of the participants identified potential barriers to uptake.

## Introduction

The presence of many moles, or melanocytic nevi, is the strongest known risk factor for melanoma [1,2]. Melanocytic nevi vary in number, size, shape, and color depending on an individual’s endogenous and exogenous factors. Studying the clinical features and changes to melanocytic nevi over time has the potential to provide greater insight into melanoma development. In 2020, almost 325,000 new cases of melanoma were detected, and melanoma resulted in nearly 60,000 deaths worldwide [3,4]. In Australia, melanoma was estimated to be the third most diagnosed cancer [5]. Survival outcomes worsen with increasing tumor thickness, and thicker melanomas require more invasive and intensive treatment; therefore, early detection is critical. For example, thin melanomas (<0.8 mm) have a 10-year survival rate of nearly 98% [6].

Total-body photography may help to confirm if nevi are stable or new and may reduce the number of nevus biopsies [7]. Recent technological advances have resulted in unprecedented changes to the landscape of dermatological photography, including the evolution from 2D photography to 3D total-body photography [8,9]. Two-dimensional images of the skin are taken and composed to form a body map, whereas 3D total-body photography allows for the collection of high-resolution macroscopic images that provide a record of almost the entire skin surface in an avatar format. Three-dimensional photography machines integrate software that presents the number, border irregularity, and color distribution of nevi and other skin lesions to the clinician, and tracking software facilitates comparison of nevi appearance over time [9,10]. Three-dimensional total-body photography allows people to view an avatar of their whole skin, including areas with sun damage, freckling, and nevus density. Furthermore, the process of 3D total-body photography takes a short time and only requires people to stand in one position, which is another advancement compared to 2D photography.

Currently 3D total-body photography is not widely available for commercial use. Few people have experienced 3D total-body photography to monitor their nevi, and no previous studies have described consumer-reported attitudes of using 3D photography in detail. The *Mind Your Moles* population-based cohort study aimed to improve the understanding of the epidemiology and biology of nevi in adults. The aim of this study was to explore the feedback provided within the *Mind Your Moles* study. This included evaluating the experience of 3D total-body photography perceived by study participants, including evaluating their level of trust and comfort toward this new technology.

## Methods

### Study Design and Participant Inclusion Criteria

This study was part of a 3-year, population-based, prospective cohort study of adults aged 20-70 years from South East Queensland, Australia. The study protocol has previously been described [11]. Participants age 20-69 years were recruited from the Australian Electoral Roll register. Participants were eligible if they had at least one nevus (any size) and Fitzpatrick skin type I to IV, and were willing to attend the clinic for 3D total-body photography every 6 months to evaluate changes in nevi over 3 years. Participants attended study visits at the Princess Alexandra Hospital, Brisbane for a clinical skin examination by a junior clinician and received 3D total-body photography and dermoscopy (VECTRA WB360 Serial Number WB00009, Canfield Scientific, Parsippany, NJ). The diagnostic process was based on the junior clinicians’ clinical examination, and if suspicious lesions were identified, a dermatologist (author HPS) reviewed the images for a second opinion. Artificial intelligence (AI) algorithms were used for providing total nevus counts over 2 mm and 5 mm.

### Ethics Approval

This study was approved by the Metro South Health Human Research Ethics Committee (approval number HREC/16/QPAH/125).

### Baseline Questionnaire

The sociodemographic characteristics of participants collected at baseline included age, sex, income, highest educational attainment, and employment status. The phenotypic and clinical characteristics collected at baseline included the innate skin color of the ventral upper arm, eye color, natural hair color at 21 years of age, BMI, and personal and familial skin cancer history.

### Participant Experience Using 3D Total-Body Photography

At the 18-month (visit four) and 36-month (final visit) follow-up visits, participants were asked to provide feedback on 3D total-body photography (see Multimedia Appendix 1). Survey questions were based on a previously developed questionnaire for consumer mobile teledermoscopy [12], adapted from the Technology Acceptance Model [13].

In optional open-ended questions, participants were asked to list benefits or disadvantages (if any) of using 3D total-body photography both at 18 months and the final visit (36 months). Two independent researchers (MO and SS) read through the complete data set twice to familiarize themselves with the qualitative data [14]. A single researcher identified broad initial themes and significant patterns within the data, which were reviewed for consistency by a second researcher. The researchers met to identify, discuss, and agree upon core themes, with any disagreements settled by a third researcher. Participant responses were tallied into themes. Participant responses that were the same at both time points (ie, the participant mentioned the same advantage or same disadvantage at both the 18-month and final visit) were combined and counted as one response. Responses that were different between time points were counted separately.

### Participant Satisfaction at the End of the Study

At the 36-month follow-up visit, participants were asked additional one-off questions with the entire photography process including if they thought it could improve the diagnosis and monitoring of skin lesions, it could be used for discovering new insights into skin well-being, they would recommend it to others, it was useful, it could help improve teaching people about their skin conditions, and it feels like an intrusion of their privacy. Participants were asked about the follow up of images including if they would like to see them at the end of the consultation, would like a copy, or would like to discuss the images with a doctor. Response options for each of these questions was *yes* or *no*. Participants were also asked their preference for the gender of the photographer (man, woman, no opinion).

### Sample Size

Sample size calculations, including the number of nevi expected for observation, have been previously reported [11]. We aimed to recruit a minimum of 188 participants to account for a 20% dropout rate, leaving a final sample size of 150 participants at the end of the follow-up period.

### Statistical Analysis

Descriptive statistics were used to present participant characteristics, experience, and satisfaction with 3D total-body photography.

The Wilcoxon matched pairs signed rank test was used to determine a change to participants’ satisfaction with 3D total-body photography between the 18- and 36-month time points (including questions surrounding comfort, trust, intended future use, and willingness to pay). Chi-square tests (or Fisher exact tests when appropriate) for categorical factors and Mann-Whitney *U* tests for continuous factors were used to assess demographic and skin health–related factors with participants’ trust and comfort with 3D total-body photography at the end of the study (36 months).

*P* values <.05 were considered statistically significant. All statistical analyses were performed in SPSS 28.0 (IBM Corp).

## Results

### Participant Characteristics

A total of 193 eligible participants participated at the baseline visit. Of these, 149 (77.2%) participants completed both the 18-month and 36-month time point patient experience questionnaire and were included in this analysis. Included participants had a median age of 55 (range 23-70) years, 63.1% (n=94) were male, and 6.7% (n=10) had previously been diagnosed with melanoma. Most participants had a fair skin type (n=116, 77.9%). Participants had a median of 49 (range 4-341) nevi >2 mm and a mean of 4 (range 0-72) nevi >5 mm (Table 1).

Attrition analysis showed that, compared to people included in the analysis, people who dropped out were mostly women (55/149, 36.9% vs 27/44, 61%; *P*=.007) and those who had fair skin (117/149, 78.5% vs 27/44, 61%; *P*=.04; data not shown).

**Table 1:** Participant characteristics (N=149).

				Participants
**Sociodemographic characteristics**				
	**Age (years)**			
		Mean (SD)	53.2 (11.5)	
		Median (range)	55 (23-70)	
		≤50 years, n (%)	54 (36.2)	
		≥51 years, n (%)	95 (63.8)	
	**Sex, n (%)**			
		Female	55 (36.9)	
		Male	94 (63.1)	
	**BMI (kg/m^2^)**			
		Mean (SD)	27.00 (4.58)	
		Median (range)	25.97 (18.36-42.75)	
		18.5-24.9 (healthy), n (%)	58 (38.9)	
		25-29.9 (overweight), n (%)	54 (36.2)	
		30 or more (obese), n (%)	35 (23.5)	
		Not reported, n (%)	2 (1.3)	
	**Combined household income (AU $)^a^, n (%)**			
		≤$39,999	16 (10.7)	
		$40,000-$79,999	29 (19.5)	
		$80,000-$124,999	31 (20.8)	
		≥$125,000	53 (35.6)	
		Unsure	8 (5.4)	
		Prefer not to answer	12 (8.1)	
	**Highest education level, n (%)**			
		University degree	66 (44.3)	
		No university degree	83 (55.7)	
	**Employment status, n (%)**			
		Full-time	74 (49.7)	
		Part-time	19 (12.8)	
		Retired	36 (24.2)	
		Other^b^	20 (13.4)	
	**Previous melanoma diagnosis, n (%)**			
		Yes	10 (6.7)	
		No	139 (93.3)	
	**Familial history of melanoma, n (%)**			
		Yes	38 (25.5)	
		No	111 (74.5)	
**Phenotypic characteristics**				
	**Skin color, n (%)**			
		Fair	116 (77.9)	
		Medium/olive	32 (21.4)	
		Not reported	1 (0.7)	
	**Natural hair color at 21 years old, n (%)**			
		Light brown	59 (39.6)	
		Fair or blonde	22 (14.8)	
		Red or auburn	6 (4.0)	
		Dark brown or black	62 (41.6)	
	**Eye color, n (%)**			
		Blue or gray	72 (48.3)	
		Green or hazel	51 (34.2)	
		Brown	25 (16.8)	
		Not reported	1 (0.7)	
	**Total nevus count >2 mm^c^**			
		Mean (SD)	68.01 (61.88)	
		Geometric mean	47.94	
		Median (range)	49 (4-341)	
	**Total nevus count >5 mm^c^**			
		Mean (SD)	7.23 (9.72)	
		Geometric mean	4.34	
		Median (range)	4 (0-72)	

^a^A currency exchange rate of AU $1=US $0.71 is applicable.

^b^Included home duties, self-employed, student, and unemployed.

^c^Total nevus counts calculated using artificial intelligence software.

### Patient Experience With 3D Total-Body Photography

Table 2 provides a summary of changes to participants’ experience of 3D total-body photography. At the 18-month time point, over two-thirds (n=103, 69.1%) of the 149 participants stated that they completely trusted 3D total-body imaging for the diagnosis and monitoring of their nevi, and this was similar at 36 months (n=104, 69.8%). Participants who reported a healthy BMI were more likely to report distrust or uncertainty toward the imaging process (10/58, 17%) compared to those with overweight or obese BMIs (5/89, 6%; *P*=.03). There was a statistically significant difference between groups of trust according to age (*P*=.04). Those who were trusting of the technology had a median age of 56 (range 23-70) years, and those who were not trusting or unsure had a median age of 48.5 (range 27-67) years.

A statistically significant difference was observed between the 18- and 36-month time points for comfort using this technology, with a reduction in the proportion of participants reporting the technology as *very comfortable* at the 36-month time point, from 71.1% (n=106/149) to 61.1% (n=91/149; *P*=.01; Table 2). Males were more likely (92/94, 98%) to report that they were more comfortable with the imaging process compared to females (48/54, 89%); however, this was only marginally significant (*P*=.05; Table 3). No other participant characteristics were associated with trust and comfort.

Almost all participants would pay a fee to use this service, and this did not change between the 18- and 36-month time points. At the end of the study, only 6.7% (n=10) of the 149 participants would not pay to use this service, 58.4% (n=87) would pay between AU $1 and AU $100, and 33.5% (n=50) would pay AU $101 or more (AU $1=US $0.71). The majority of participants (140/149, 94%) would consider using the technology in the future if it were commercially available with their regular medical practitioner, and this did not change between the 18- and 36-month time points.

A total of 149 participants provided a response to the open-ended question about advantages of 3D total-body photography (Table 4). Six key themes emerged, including (1) comprehensive skin check and early detection; (2) improved monitoring; (3) satisfaction, time efficiency, and improved health output; (4) noninvasive procedure; (5) accuracy and AI; and (6) contribution to research (altruism).

Many of the 149 participants (n=95, 63.8%) stated that the technology provided a “Comprehensive overview of all the body surface.” Participants (n=88, 59.1%) were positive about 3D total-body photography providing an accurate baseline to record, compare, and follow changes in their skin over time. Over one-tenth (n=16, 10.7%) of participants described the process of 3D total-body photography as “painless,” “non-intrusive,” and “less invasive than [a] regular skin check procedure.”

Half (n=74, 49.7%) of the 149 participants reported disadvantages of 3D total-body photography. Four key themes emerged, including (1) physical privacy, (2) travel, (3) concerns about new technology, and (4) cost. In terms of physical privacy, 20 (13.4%) participants stated they were “not comfortable in underwear” and disliked “feeling exposed.” One-fifth (n=30, 20.1%) of participants expressed concerns regarding the new technology, including its ability to accurately detect suspicious lesions and body areas unable to be imaged, and 3 participants mentioned concerns about digital security.

**Table 2:** Patient experience of using 3D total-body photography at 18- and 36-month follow-up visits (N=149).

Question			Time point, n (%)					*P* value
			18-month		36-month			
**How much do you trust this 3D total-body photography for the diagnosis and monitoring of your moles?**								.68
	Completely trust	103 (69.1)		104 (69.8)				
	Slightly trust	26 (17.4)		29 (19.5)				
	Unsure/slightly/completely do not trust	16 (10.7)		16 (10.7)				
	Not reported	4 (2.7)		0 (0.0)				
**How comfortable were you in participating in the 3D total-body photography?**								.01
	Very comfortable	106 (71.1)		91 (61.1)				
	Comfortable	32 (21.5)		49 (32.9)				
	Indifferent	2 (1.3)		3 (2.0)				
	Slightly not comfortable	5 (3.4)		5 (3.4)				
	Not at all comfortable	0 (0.0)		0 (0.0)				
	Not reported	4 (2.7)		1 (0.7)				
**Would you consider using 3D total-body photography if it becomes commercially available with your regular medical practitioner?**								.71
	Yes	140 (94.0)		141 (94.6)				
	No	4 (2.7)		6 (4.0)				
	Not reported	5 (3.4)		2 (1.3)				
**How much would you be willing to spend on this service if it became available at your dermatologist’s practice? (AU $)^a^**								.68
	$0	6 (4.0)		10 (6.7)				
	$1 to $50	31 (20.8)		27 (18.1)				
	$51 to $100	64 (43.0)		60 (40.3)				
	$101 to $200	37 (24.8)		40 (26.8)				
	$201 or more	6 (4.0)		10 (6.7)				
	Not reported	5 (3.4)		2 (1.3)				

^a^A currency exchange rate of AU $1=US $0.71 is applicable.

**Table 3:** Patient characteristics associated with patient trust and comfort at the end (36 months) of the intervention.

Demographic characteristics		Trust^a^ (N=149)					Comfort^b^ (n=148)				
		Yes (n=133)	Unsure/no (n=16)	*P* value		Yes (n=140)		Unsure/no (n=8)		*P* value	
**Age (years)**					.10						.14
	≤50, n (%)	45 (83.3)	9 (16.7)			48 (90.6)		5 (9.4)			
	≥51, n (%)	88 (92.6)	7 (7.4)			92 (96.8)		3 (3.2)			
	Median (range)	56 (23-70)	48.5 (27-67)	*.04* ^c^		55 (23-70)		47 (26-64)		.39	
**Sex, n (%)**					.59						.05
	Female	48 (87.3)	7 (12.7)			48 (88.9)		6 (11.4)			
	Male	85 (90.4)	9 (9.6)			92 (97.9)		2 (2.1)			
**Highest education level, n (%)**					.18						.47
	University degree	56 (84.8)	10 (15.2)			61 (92.4)		5 (7.6)			
	No university degree^d^	77 (92.8)	6 (7.2)			79 (96.3)		3 (3.7)			
**Personal history of melanoma, n (%)**					.99						.99
	Yes	9 (90.0)	1 (10.0)			10 (100.0)		0 (0.0			
	No	124 (89.2)	15 (10.8)			130 (94.2)		8 (5.8)			
**Family history of melanoma, n (%)**					.12						.99
	Yes	31 (81.6)	7 (18.4)			35 (94.6)		2 (5.4)			
	No	102 (91.9)	9 (8.1)			105 (94.6)		6 (5.4)			
**Skin color^e^, n (%)**					.99						.69
	Fair	103 (88.8)	13 (11.2)			108 (93.9)		7 (6.1)			
	Medium/olive	29 (90.6)	3 (9.4)			31 (96.9)		1 (3.1)			
**BMI^e^ (kg/m^2^)**					*.03*						.44
	Healthy, n (%)	48 (82.8)	10 (17.2)			54 (93.1)		4 (6.9)			
	Overweight/obese, n (%)	84 (94.4)	5 (5.6)			85 (96.6)		3 (3.4)			
	Median (range)	26.2 (18.3-42.7)	23.4 (19.5-34.5)	.06		26.0 (18.3-42.7)		25.0 (21.1-34.9)		.78	
**Total nevus count >2 mm**					.86						.34
	0-19, n (%)	21 (95.5)	1 (4.5)			19 (90.5)		2 (9.5)			
	20-49, n (%)	46 (85.2)	8 (14.8)			50 (92.6)		4 (7.4)			
	≥50, n (%)	66 (90.4)	7 (9.6)			71 (97.3)		2 (2.7)			
	Median (range)	49 (4-341)	43.5 (8-266)	.90		50 (4-332)		38.5 (17-341)		.60	
**Total naevus count >5 mm**					.72						.63
	0-4, n (%)	72 (91.1)	7 (8.9)			74 (93.7)		5 (6.3)			
	5-10, n (%)	36 (87.8)	5 (12.2)			39 (97.5)		1 (2.5)			
	≥20, n (%)	25 (86.2)	4 (13.8)			27 (93.1)		2 (6.9)			
	Median (range)	4 (0-72)	6.5 (0-67)	.87		4 (0-36)		3.5 (2-72)		.59	

^a^Completely trust and slightly trust were combined into a single category of trust, and unsure, slightly do not trust, or completely do not trust were combined into a single category of distrust.

^b^Very comfortable and comfortable were combined into a single category of comfort, and indifferent and slightly not comfortable were combined into a single category of discomfort.

^c^Italics indicate that the *P* value is significant at the .05 level.

^d^No university degree includes those who completed secondary school, certificate, diploma, trade, or apprenticeship.

^e^Data missing: 1 participant’s skin type and BMI.

**Table 4:** Qualitative feedback from participants on the advantages and disadvantages of total 3D total-body photography to monitor nevi.

		Participants (N=149), n (%)	Example
**Advantages (themes)**			
	Comprehensive skin check and early detection	95 (63.8)	“It covers every angle of the skin, comprehensive system, if anything is observed, it is sent to a specialist for review...can be followed up and acted on quickly.” (male, 45 years old) “Gives a clear picture of entire body.” (male, 66 years old)
	Improved monitoring (follow and record changes to skin over time; improved awareness and self-management)	88 (59.1)	“Additional reassurance to have a thorough skin examination, kept on record, can be reviewed, can monitor changes over time.” (male, 58 years old) “Excellent way of tracking your moles, having a baseline for assessment.” (male, 45 years old) “Peace of mind, having a photographic record means you can track changes over time.” (male, 61 years old)
	Satisfaction, time efficiency, and improved health output	64 (43.0)	“Fantastic, quicker to take photos, more detail from patient photos, overall great idea.” (female, 56 years old) “Time saving, maybe don't need a Doctor to check every single spot at time of appointment. Patient more likely to have skin checked if time efficient process, like VECTRA.” (female, 37 years old)
	Noninvasive procedure	16 (10.7)	“Painless, not intrusive.” (male, 53 years old) “Simple process, non-invasive, comprehensive reference to look back on.” (male, 58 years old)
	Accuracy and artificial intelligence	33 (22.1)	“Accuracy and precise - shows the whole body. Exciting new technology.” (female, 64 years old) “More preci[se], up to date technology, not just human only assessment of skin.” (female, 52 years old)
	Contribution to research (altruism)	23 (15.4)	“To assist in research to benefit future generations.” (female, 66 years old) “Research towards future diagnosis of melanoma.” (male, 52 years old)
	No comments	5 (3.4)	N/A^a^
**Disadvantages (themes)**			
	Physical privacy (body image, self-conscious)	20 (13.4)	“Undressing in front of strangers.” (female, 38 years old) “Looking at your body in 3D is confronting...” (female, 46 years old) “Not everyone is comfortable taking [their] clothes off.” (male, 32 years old)
	Concerns about new technology (trust, ability to accurately detect suspicious lesions, digital security, and privacy)	30 (20.1)	“[I] wouldn't trust 3D total-body photography without having a trained clinician present to look at [my] skin and/or review the images.” (male, 45 years old) “Do still need the naked eye. Doesn’t take away the need for a human.” (female, 34 years old) “Should always be complimented by a doctor looking at the skin.” (male, 65 years old) “Human eye gives a more complete view of the whole body. Some areas are missed by VECTRA (scalp, soles of feet).” (male, 44 years old) “Knowing there are all these identifiable photos of you stored.” (female, 36 years old)
	Travel (accessibility to machine)	14 (9.4)	“Having to come into the hospital to do it. It is a big machine, so would not be able to have one in many locations.” (female, 29 years old) “One location at PA [Princess Alexandra Hospital], way to travel.” (female, 48 years old) “The time cost of travelling to the machine.” (male, 66 years old)
	Cost	10 (6.7)	“If there was a cost associated with [3D body imaging], depending on the magnitude...” (male, 58 years old) “Machine expensive.” (male, 62 years old)
	No disadvantages identified	75 (50.3)	N/A

^a^N/A: not applicable.

### Participant Satisfaction at the End of the Study

Table 5 reports participants’ satisfaction with the 3D total-body photography processes, with 2 to 3 participants opting to not answer one or more of these questions. At the conclusion of the 36-month study, most participants (146/147, 99.3%) perceived 3D total-body photography to be a useful tool. The predominant belief was that this technology can improve the diagnosis and monitoring of skin lesions (147/148, 99.3%), with only 1 participant disagreeing. Only 2% (3/148) of participants would not recommend 3D total-body photograph to others. While most participants (130/148, 87.8%) had no preference for the gender of the photographer, no participant indicated preference for a male photographer. Following 3D total-body photography visits, most participants (115/146, 78.8%) wanted to discuss the images with a doctor, and 47.6% (70/147) wanted a copy of the images.

**Table 5:** Participant satisfaction with 3D total-body imaging at the 36-month time point (n=148).

Question		Participants, n (%)
**It can improve diagnosis and monitoring of skin lesions**		
	Yes	147 (99.3)
	No	1 (0.7)
**It can be used for discovering new insights into skin well-being**		
	Yes	145 (98.0)
	No	3 (2.0)
**It feels like an intrusion on your privacy**		
	Yes	9 (6.1)
	No	139 (93.9)
**Would you rather be photographed by a...**		
	Man	0 (0.0)
	Woman	18 (12.2)
	No opinion	130 (87.8)
**Would you recommend total 3D body photography to your friends and family?**		
	Yes	145 (98.0)
	No	3 (2.0)
**It is useful (n=147)**		
	Yes	146 (99.3)
	No	1 (0.7)
**It can improve teaching people about their skin conditions (n=147)**		
	Yes	143 (97.3)
	No	4 (2.7)
**Would you like to see the images at the end of the consultation (n=147)**		
	Yes	110 (74.8)
	No	37 (25.2)
**Would you like to have a copy of the images (n=147)**		
	Yes	70 (47.6)
	No	77 (52.4)
**Would you like to discuss the images with a doctor (n=146)**		
	Yes	115 (78.8)
	No	31 (21.2)

## Discussion

### Principal Findings

This study explored participants’ experiences of 3D total-body photography. The majority of participants were comfortable and trusted the imaging process at both the 18- and 36-month time points. Results also showed almost all participants would pay a fee to use this service in the future and would recommend it to others. Furthermore, most participants thought it could improve diagnosis and monitoring of skin lesions. While high levels of satisfaction were reported, when asked to list barriers, half of the participants identified one or more including trust, privacy, cost, and travel requirements. The feedback collected in this study is important, as perceived usefulness and ease of use are essential constructs for the adoption of new technologies [13,15,16].

Developing and implementing a new medical device or technology requires insight into consumer preferences to ensure that the service is used. While 3D total-body photography is practiced in Australia, it is done so primarily in research settings and in an informal manner in practical settings. Total-body photography has been found to result in detection of a higher proportion of in situ melanomas and thin invasive melanomas compared to consults without total-body photography [17]. The exact localization of suspicious lesions is particularly useful in the clinical setting to enable accurate follow-up [17]. This study has the potential to assist with the translation and implementation of 3D total-body photography from the current informal provision into a formal service or screening program. Understanding the participant experience of 3D total-body photography allows researchers to identify which aspects are working (benefits) and which aspects are not working or suboptimal (barriers) in a research setting, and this information can help to identify areas for improvement in the clinical setting. Here, we found the benefits included a comprehensive record of the skin to allow improved monitoring, while the main barrier identified was trust, followed by privacy, cost, and travel requirements.

Issues surrounding trust are well known when researching new technological innovations in health care and have been previously reported in other studies assessing melanoma imaging [12,18,19]. Only 11% (n=16/149) of participants did not trust the imaging process at the end of the study. Two-thirds of these participants were those with a healthy BMI and younger than 50 years (Table 3). Previous research suggests that people may be less trusting and accepting of automation in health care settings compared to other aspects of life such as transport [20]. However, other research reported strong support for the use of automated diagnostic tools if clinicians continue to assess patients independently as well [20]. This emphasizes the value people assign to their doctor-patient relationships and suggests that clinicians will play a pivotal role in the acceptance of 3D total-body imaging as a part of routine practice. Clinicians have also cited barriers to using total-body photography including the belief that it may lead to more biopsies or greater patient anxiety, as well as logistical constraints such as lack of time, availability, training, and associated costs [21]. We foresee that the introduction of AI into dermatology will present further challenges for trust of telemedicine services in both consumers and clinicians. In this study, the AI algorithms only provided the clinicians with a count of skin lesions and sorted them by size, color, and border irregularity. In the future, AI may assist clinicians in deciding what type of skin lesions are present, with some studies suggesting that AI can perform similarly to dermatologists, but further validation in practice is required [22-24]. Future studies are warranted to examine trust with health care technologies using AI.

We found that the majority of the participants were satisfied with this technology, and there were some differences in satisfaction based on gender, age, and BMI. The factors identified may help to create a profile of patients who would require attention to ensure adequate uptake of the technology. We found males were more likely to be comfortable with the imaging process than their female counterparts. Some females reported in the qualitative comments that undressing in front of strangers and looking at their body in the images could be confronting. In addition, after further analysis of the 18 participants who would prefer to be photographed by a woman, 16 were female (data not shown). Overall, we found participants in this study showed high engagement by wanting to discuss the images with their doctor, and this could be used as a potential learning opportunity in the future for clinicians to show their patients what changes to look for to further support monitoring and early detection efforts.

### Strengths and Limitations

This prospective cohort study recruited participants from a population-based registry and achieved a good number of participants who completed both the 18- and 36-month time point questionnaires to provide feedback on 3D total-body photography (149/193, 77.2%), providing a rich data set of their attitudes and changes over time.

The limitations include that the data were self-reported and subject to potential biases (eg, recall bias and socially desirable responses). This study involved participants having regular skin checks, and therefore, the volunteer sample might be more motivated and accepting of this technology. Just under half the participants in the study had a high level of education.

Participants were first asked about their experiences of 3D total-body photography at the fourth photography session after using the technology several times. Asking participants about their views prior to use at baseline would have allowed greater insights into how participant views changed over time.

### Conclusion

Participants from the general population supported the use of 3D total-body photography for the monitoring of their nevi; albeit, half had some concerns regarding the technology. Consultation with participants and understanding their experience using the new technology will be important for the future translation of 3D total-body photography into standard dermatological care.

## Appendix

Multimedia Appendix 1 The 3D total-body imaging questionnaire.

## Abbreviations

AI: artificial intelligence
NHMRC: National Health and Medical Research Council
