# Supplementary material for: The Experience of 3D Total-Body Photography to Monitor Nevi: Results From an Australian General Population-Based Cohort Study
Source: JMIR Dermatol. 2022 Jun 20;5(2):e37034. doi: 10.2196/37034 (PMC10334884; doi:10.2196/37034)
Supplement: Multimedia Appendix 1 [file derma_v5i2e37034_app1.pdf]

VECTRA Feedback

Record ID __________________________________

Not many people have experienced total body 3D photography using the VECTRA to monitor their naevi. Hence we would like feedback on the use of the VECTRA and how it made people feel to better inform us on future use in clinical practice.
Please note there are no right or wrong answers it's all about your personal views and experiences.

In your view, what are the benefits of total 3D body photography using the VECTRA? ____

In your view, what are the disadvantages of total 3D body photography using the VECTRA (if any)? ____

How effective do you believe total body 3D imaging was at monitoring changes in your moles?
- ◯ Very effective
- ◯ Effective
- ◯ Unsure
- ◯ Slightly not effective
- ◯ Not at all effective

How much do you trust this total body 3D imaging for the diagnosis and monitoring of your moles?
- ◯ Completely trust
- ◯ Slightly trust
- ◯ Unsure
- ◯ Slightly do not trust
- ◯ Completely do not trust

How comfortable were you in participating in the total 3D body photography?
- ◯ Very comfortable
- ◯ Comfortable
- ◯ Indifferent
- ◯ Slightly not comfortable
- ◯ Not at all comfortable

Can you please describe a bit more on what makes you feel comfortable? ____

Can you please describe a bit more on what makes you feel not comfortable? ____

Would you consider using the VECTRA if it becomes commercially available with your regular medical practitioner?
- ◯ Yes
- ◯ No

Please specify why not ____

How much would you be willing to spend on this service if it became available at your dermatologist's practice?	◯ $0<br>◯ $0 to $50<br>◯ $51 to $100<br>◯ $101 to $200<br>◯ $201 or more
Please provide us with any other feedback you may have in regards to your experience with the total body 3D photography:	__________________________________

Exit Survey

Please complete the survey below for the Mind your Moles study

Thank you for participating in the Mind your Moles study and helping us fight against melanoma!

1) How comfortable were you in participating in the 3D total body photography?
   - ◯ Very comfortable
   - ◯ Comfortable
   - ◯ Indifferent
   - ◯ Slightly not comfortable
   - ◯ Not at all comfortable

2) If you stated that you were comfortable participating in the 3D total body photography, please describe what makes you feel comfortable: ______________________________

3) If you stated that you were not comfortable participating in the 3D total body photography, please describe what makes you feel uncomfortable: ______________________________

4) How much do you trust total body imaging for the diagnosis and monitoring of your moles?
   - ◯ Completely trust
   - ◯ Slightly trust
   - ◯ Unsure
   - ◯ Slightly do not trust
   - ◯ Completely do not trust

Do you agree with the following statements regarding 3D total body photography:

		Yes	No
5)	It is useful	◯	◯
6)	It can improve diagnosis and monitoring of skin lesions	◯	◯
7)	It can improve teaching people about their skin conditions	◯	◯
8)	It can be used for discovering new insights into skin wellbeing	◯	◯
9)	It feels like an intrusion on your privacy	◯	◯
10)	It can make you feel uncomfortable	◯	◯

11) In your view, what are the benefits of total 3D body photography using the VECTRA? ______________________________

12) In your view, what are the disadvantages of total 3D body photography using the VECTRA? ______________________________

13) Would you rather be photographed by a:
   - ◯ Man
   - ◯ Woman
   - ◯ No opinion

Would you like to:

	Yes	No
14) See the images at the end of the consultation?	◯	◯
15) Discuss the images with a doctor?	◯	◯
16) Have a copy of the images?	◯	◯

17) Would you consider using VECTRA if it becomes commercially available with your regular medical practitioner?
◯ Yes
◯ No

18) How much would you be willing to spend on this service?
◯ $0
◯ $1 - 50
◯ $51 - 100
◯ $101 - 200
◯ $201 or more

19) Would you recommend total 3D body photography to your friends and family?
◯ Yes
◯ No

20) Please provide us with any other feedback you may have in regards to your experience with the total 3D photography:
____

21) Would you like to be contacted about participating in future studies related to melanoma imaging?
◯ Yes
◯ No
